# First-Trimester Serum Cytokine Profile in Pregnancies Conceived After Assisted Reproductive Technology (ART) With Subsequent Pregnancy-Induced Hypertension

**DOI:** 10.3389/fimmu.2022.930582

**Published:** 2022-07-01

**Authors:** Xiangxin Lan, Ling Guo, Shiqin Zhu, Yongzhi Cao, Yue Niu, Shuwen Han, Zeyan Li, Yan Li, Junhao Yan

**Affiliations:** ^1^ Center for Reproductive Medicine, Shandong University, Jinan, China; ^2^ Key Laboratory of Reproductive Endocrinology of Ministry of Education, Shandong University, Jinan, China; ^3^ Medical Integration and Practice Center, Shandong University, Jinan, China; ^4^ School of Biomedical Sciences, Shandong University, Jinan, China; ^5^ Suzhou Research Institute, Shandong University, Suzhou, China

**Keywords:** cytokine profile, first trimester of pregnancy, pregnancy-induced hypertension, assisted reproductive technology, biomarker

## Abstract

Pregnancy-induced hypertension (PIH) is one of the most common pregnancy complications that seriously affects the mother and fetus. The incidence of PIH is higher in pregnancies conceived after assisted reproductive technology (ART) than in spontaneous pregnancies; thus, exploring potential serum biomarkers before PIH onset is of great significance for effective early prediction and prevention of PIH in the ART population. Cytokines are involved in the inflammatory response and immune regulation, which play an essential role in the pathogenesis of PIH. A description of the cytokine profile in the first trimester of pregnancy could help identify new diagnostic tools and develop targeted therapies for PIH in the ART population. The concentrations of classical predictive markers for PIH and another 48 cytokines were measured in the first-trimester pregnancy serum samples from 33 PIH patients and 33 matched normotensive controls (NC), both of whom conceived after ART treatment. The measured values were compared and analyzed between NC and PIH, followed by comprehensive bioinformatic analysis and logistic regression analysis. There was no significant difference in classical predictive markers, including Activin A, PlGF, sFLT1 (VEGFR), and sFLT1/PlGF, between the PIH and NC groups (*P* > 0.05), while 29 cytokines were significantly lower in the PIH group than in the NC group (*P* < 0.05). Logistic regression analysis revealed that 17 cytokines (IL-2Rα, M-CSF, IL-6, IL-2, β-NGF, IL-7, IL-12 (p70), SCF, IL-10, IL-9, MIG, GM-CSF, LIF, IL-1α, MCP-3, IL-4, and HGF) in the first-trimester pregnancy serum were significantly negatively correlated with the subsequent onset of PIH. With the top 3 cytokines (IL-7, MIG, and SCF) of receiver operating characteristic (ROC) analysis, we constructed an efficient multifactor combined detection and prediction model for PIH in ART pregnancy. Classical early predictors for hypertensive disorder complicating pregnancy cannot distinguish PIH from their normal peers in ART pregnancy. In comparison, the description of the cytokine profile in the first trimester of pregnancy enables us to distinguish high-risk ART pregnancy for PIH, permitting enough time for PIH prevention therapy. The cytokine profile we described also provides immunological insight into the further mechanistic exploration of PIH.

## Introduction

Pregnancy-induced hypertension (PIH) is one of the most common pregnancy complications, affecting 6% to 10% of pregnancies (1, 2). It is defined as a new onset of hypertension (systolic pressure ≥ 140 mmHg and/or diastolic pressure ≥ 90 mmHg) with or without proteinuria after the 20th week of gestation, which includes preeclampsia (3). PIH is characterized by maternal hypertension and multiorgan involvement resulting from systemic endothelial dysfunction, and it is also a major cause of maternal and fetal morbidity and mortality (4, 5). However, the clinical intervention of PIH stagnates in passive treatment after symptoms appear, resulting in poor prognosis. Hence, early prediction markers for PIH are of great significance for presymptomatic prophylaxis.

To date, there are several well-studied molecular markers related to placental hypoperfusion, such as placental growth factor (PlGF), soluble Fms⁃like tyrosine kinase-1 (sFlt-1) or vascular endothelial growth factor receptor (VEGFR) and Activin A. Although a high sFlt-1:PlGF ratio (6–8), high Activin A serum level (9) and low PlGF serum level (8) have been proven to be able to separate PIH from normotensive pregnant women, they all share low predictive accuracy in the first trimester of pregnancy (10, 11). Unfortunately, it has recently been found that the incidence of PIH in the ART population is higher than that in spontaneous pregnancy (12). Guidelines suggest that oral aspirin should be started at 11-14^+6^ gestational weeks in high-risk populations to achieve effective prevention (13). Therefore, it is urgent to explore biomarkers for the first-trimester prediction of PIH in the ART population.

The pathophysiological mechanisms of the development of PIH remain unclear. One of the more deeply studied mechanisms is fetal-maternal immunity (14). There is increasing evidence that PIH may originate from poor maternal immune tolerance and abnormal interactions between immune cells, trophoblast cells, and decidual stromal cells after embryo implantation. Immune cells such as natural killer cells, dendritic cells, and T regulatory lymphocytes in the decidua have impaired immune tolerance to spiral artery remodeling and the emergence of fetal trophoblasts, leading to increased placental shedding, exaggerated systemic inflammation, and subsequent endothelial dysfunction (15).

Cytokines are a family of small soluble proteins expressed by various cell and tissue types that serve as immune mediators. Their expression profiles have been used to classify immune responses and functional states of the immune system. Evidence suggests that cytokines play a crucial role in ovulation, implantation, placentation, and parturition during pregnancy (16, 17). Recent research indicates that PIH is related to immune dysregulation and that components of the immune system may interact with angiogenic and antiangiogenic factors (12). In this study, the cytokine profiles were characterized in paired PIH and normotensive women following ART treatment to determine the specific cytokine signature involved, which would be of great value to explain the occurrence of PIH after ART and provide new avenues for early diagnosis, monitoring, and intervention.

## Materials and Methods

### Participants

In this study, 33 women diagnosed with PIH and 33 controls were selected from patients who underwent *in vitro* fertilization (IVF)/intracytoplasmic sperm injection (ICSI) cycles based on a large prospective cohort conducted at the Research Center of the Reproductive Hospital Affiliated to Shandong University from January 2018 to June 2019. All patients were < 40 years and achieved singleton pregnancy by assisted reproductive technology in this pregnancy. Patients diagnosed with chronic autoimmune disease (such as systemic lupus erythematosus, thyroid autoimmunity, or antiphospholipid syndrome), preexisting secondary/essential hypertension, gestational diabetes mellitus, and other diseases that may affect the inflammatory process were excluded. Pregnancies after embryo transfer were followed by periodic reviews of electronic medical records until delivery. PIH was defined as a new onset of hypertension (systolic pressure ≥ 140 mmHg and/or diastolic pressure ≥ 90 mmHg) with or without proteinuria after the 20th week of gestation. According to clinical and laboratory evaluations, 33 patients diagnosed with PIH were included in the PIH group. To achieve a balanced baseline characteristics, propensity score matching (PSM) was applied to control for potential confounders, including age, BMI, cause of infertility, fertilization method, and embryo transfer proposal, which were weighted equally. The NC group included 33 healthy women who were matched in a 1:1 ratio to PIH based on the propensity score with a standard caliper width of 0.1. This study protocol was approved by the Ethical Committee of Reproductive Medicine of Shandong University, and all participants signed informed consent forms.

### Sample Collection

At 11-13 gestational weeks following IVF/ICSI, 6 ml fasting blood samples of the participants were collected by venipuncture. After centrifugation at 1300 × g for 10 minutes, serum samples were separated and stored at −80°C until measurement.

### ELISA Analysis

The serum levels of Activin A, PlGF and sFLT1 were measured using commercial ELISA kits (R&D, Cat # DAC00B, Human/Mouse/Rat Activin A Quantikine ELISA Kit; Cat # DPG00, Human PLGF Quantikine ELISA Kit; Cat # DVR100C, Human VEGFR1/Flt-1 Quantikine ELISA Kit).

### Cytokine Profiling

Individual serum samples were subjected to cytokine profile measurement by the Bio-Plex Pro Human Cytokine Screening Panel, 48-plex (BioRad). The protocol of the kit was followed for the analysis of a total of 48 cytokines, chemokines or growth factors. These included interleukin-1β (IL-1β), IL-1α, IL-1ra, IL-2, IL-2Rα, IL-3, IL-4, IL-5, IL-6, IL-7, IL-8, IL-9, IL-10, IL-12 (p40), IL-12 (p70), IL-13, IL-15, IL-16, IL-17A, IL-18, interferon-α2 (IFN-α2), interferon-γ (IFN-γ), tumor necrosis factor-α (TNF-α), tumor necrosis factor-β (TNF-β), granulocyte colony-stimulating factor (G-CSF), macrophage colony-stimulating factor (M-CSF), granulocyte-macrophage colony-stimulating factor (GM-CSF), leukemia inhibitory factor (LIF), stem cell factor (SCF), vascular endothelial growth factor (VEGF), Eotaxin, macrophage inflammatory protein-1α (MIP-1α), macrophage inflammatory protein-1β (MIP-1β), basic fibroblast growth factor (FGF basic), monocyte chemotactic protein-1 (MCP-1) or monocyte chemoattractant activating factor (MCAF), monocyte chemotactic protein-3 (MCP-3), nerve growth factor-β (β-NGF), RANTES, stromal cell derived factor-1α (SDF-1α), platelet-derived growth factor-BB (PDGF-BB), growth related oncogene-α (GRO-α), hepatocyte growth factor (HGF), interferon inducible protein-10 (IP-10), cutaneous T-cell attracting chemokine (CTACK), mifepristone (MIF), gamma-interferon-induced monokine (MIG), stem cell growth factor-β (SCGF-β), and TNF-related apoptosis-inducing ligand (TRAIL).

### Statistical Analysis

Statistical analysis and graphical representations were completed in IBM SPSS Statistics 21 and GraphPad Prism. Normality was assessed by Kolmogorov–Smirnov tests. Normally distributed data are presented as the mean ± SEM, and the t test was used to compare the significant differences between groups. For data with a nonnormal distribution, the median with interquartile range was applied, and the Mann–Whitney U test was used for data comparison. Values of *P* < 0.05 were considered statistically significant. Principal component analysis (PCA), heatmap and cluster analysis, forest map, and half-violin plots were conducted using the website www.bioinformatics.com.cn. Univariate logistic regression analysis was performed to assess the longitudinal association between first-trimester serum cytokine levels and the occurrence of PIH. OR > 1 indicates a risk factor, and OR < 1 indicates a protective factor. Cytokines with *P* < 0.05 were considered to be related to the occurrence of PIH and included in Receiver operating characteristic (ROC) analysis. ROC curves were drawn to analyze the independent predictive value of specific cytokines for PIH. Additionally, IL-7, MIG, and SCF were selected to build a combined prediction model, and the combined diagnostic value of the prediction model for PIH was analyzed.

## Results

### Baseline Characteristics and Perinatal Outcomes of the Participants

**Table 1** displays the baseline characteristics and perinatal outcomes of 33 women who developed PIH in later pregnancy and their 33 matched normotensive controls (NC). The systolic pressure (NC 115.48 ± 2.15 vs. PIH 127.07 ± 2.17, *P* < 0.01) and mean arterial pressure (NC 85.30 ± 1.86 vs. PIH 92.66 ± 1.83, *P* = 0.01) before pregnancy were higher in the PIH group than in the NC group. There were no significant differences between the two groups in other prepregnancy baseline characteristics. The incidence of cesarean section was significantly higher in the PIH group than in the NC group (NC 66.70% vs. PIH 96.97%, *P* < 0.01). The gestational age at delivery was lower in the PIH group than in the NC group (NC 275 days vs. PIH 269 days, *P* = 0.02). Lower gestational age and higher cesarean section rate are related to each other and exactly reflect the characteristics of PIH.

**Table 1:** Baseline characteristics and perinatal outcomes of the participants.

		NC (N = 33)	PIH (N = 33)	P Value
**Prepregnancy baseline characteristics**				
Age (yr)-Mean ± SD		30.21 ± 4.29	29.94 ± 3.75	0.78
BMI (kg/m²)-Mean ± SD		25.17 ± 3.92	25.21 ± 3.94	0.97
Blood pressure (mmHg)-Mean ± SD				
	Systolic pressure	115.48 ± 2.15	127.07 ± 2.17	< 0.01*
	Diastolic pressure	70.21 ± 1.90	75.45 ± 1.87	0.06
	Mean arterial pressure	85.30 ± 1.86	92.66 ± 1.83	0.01*
Primigravida-no. (%)		29 (87.88)	29 (87.88)	> 0.99
PCOS-no. (%)		6 (46.2)	7 (53.8)	0.80
Cause of infertility-no. (%)				0.96
	Pelvic factor	10 (30.30)	9 (27.27)	
	Male factor	2 (6.06)	2 (6.06)	
	Confounding factor	21 (63.64)	22 (66.67)	
Endometrial preparation protocol-no. (%)				0.80
	Natural cycle	14 (42.42)	13 (39.39)	
	Hormonally controlled	19 (57.58)	20 (60.61)	
Mode of fertilization-no. (%)				0.60
	IVF	21 (63.64)	23 (69.70)	
	ICSI	12 (36.36)	10 (30.30)	
**Perinatal outcomes**				
Delivery mode-no. (%)				< 0.01*
	Vaginal delivery	11 (33.33)	1 (3.03)	
	Cesarean delivery	22 (66.67)	32 (96.97)	
Gestational age at delivery (day)-Median (P25, P75)		275.00 (269.00,279.50)	269.00 (263.50, 273.00)	0.02*
Delivery at <37 wk-no. (%)		4 (12.12)	5 (15.15)	> 0.99
Neonatal birth weight (g)-Mean ± SD		3319.09 ± 107.72	3137.19 ± 93.48	0.21
Low birth weight infants (<2500 g)-no. (%)		3 (9.09)	2 (6.06)	0.64

Values are the mean ± SD/median (P25, P75) or n. (%). BMI, body mass index; PCOS, polycystic ovarian syndrome; IVF, in vitro fertilization; ICSI, intracytoplasmic sperm injection. Significant difference (P < 0.05, marked by *).

### Classical Serum Biomarker Levels in Pregnancy After Assisted Reproductive Technology

First, we wanted to verify whether classical serum biomarkers can distinguish PIH from NC in ART pregnancy. Activin A, PlGF and sFLT1 levels tested by ELISA and accordingly calculated sFlt1/PlGF are shown in **Table 2**. In the first-trimester serum of ART pregnancy, classical biomarkers appeared to have no predictive validity with no significant difference between NC and the women who developed PIH later in pregnancy.

**Table 2:** Classical serum biomarker levels in pregnancy after assisted reproductive technology.

Cytokine	NC (N = 33)	PIH (N = 33)	*P* Value
Activin A (pg/mL)	2168.54 (1247.21, 3105.89)	1917.64 (1340.23, 2754.65)	0.621
PlGF (pg/mL)	56.43 (43.23, 71.14)	44.48 (36.06, 71.30)	0.221
sFlt1 (VEGFR) (pg/mL)	14401.52 (9859.96, 19593.74)	11592.32 (7951.62, 14677.62)	0.052
sFlt1/PlGF	243.43 (178.763, 315.70)	229.72 (163.65, 314.83)	0.485

Values are median (P25, P75). PlGF, placental growth factor; sFLT1, soluble fms-like tyrosine kinase-1. Significant difference (P < 0.05).

### Cytokine Profile in First-Trimester Serum in ART Pregnancy

**Figure 1:**
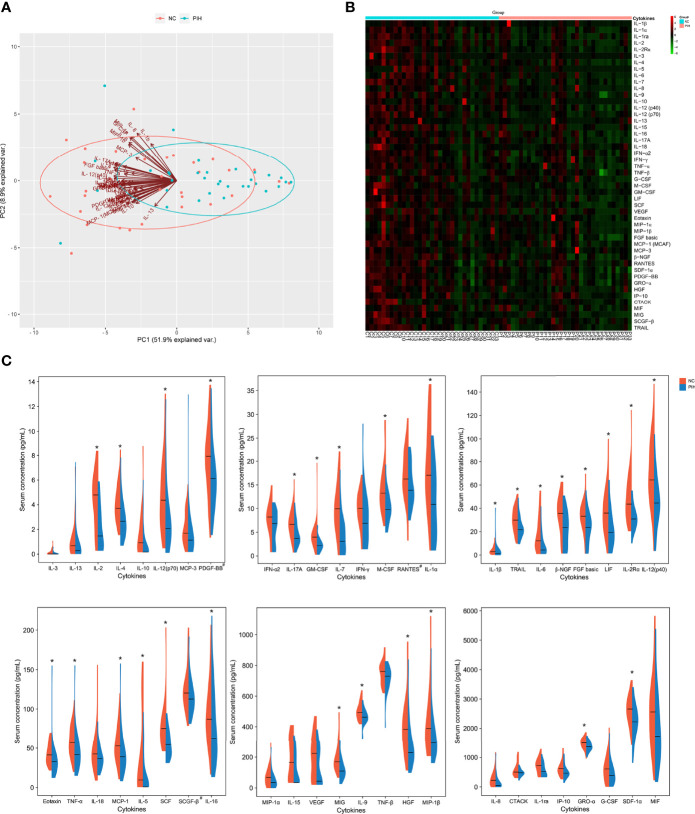
Analysis of 48 cytokine profiles in the first-trimester pregnancy serum of the NC group vs. the PIH group. **(A)**, PCA of 48 cytokine profiles in the first-trimester pregnancy serum. The points in the diagram represent the samples, red for the NC group and blue for the PIH group. The arrows represent the contribution and correlation of the corresponding original variables to the principal component. **(B)**, Heatmap analysis of 48 cytokine profiles in the first-trimester pregnancy serum. The row displays the cytokines, and the column represents the sample number. The color key represents the deviation of global median concentrations for each cytokine. These median values (homogenized to 0 and displayed in dark) were calculated after homogenization treatment and used as a cut-off to separate high and low cytokine concentrations. Color gradation from red to green indicates high to low cytokine concentrations, respectively, with homogenized values ranging from 6 to -6. **(C)**, Half-violin plots showing the expression distributions of 48 cytokines in the first-trimester pregnancy serum between the NC group and the PIH group. Six boxes were plotted according to general cytokine concentrations. The ordinate indicates the serum concentration of cytokines, with pg/mL as the concentration unit, except for the concentration units of PDGF-BB, RANTES, and SCGF-β (marked by #) being ng/mL. Every different cytokine is shown as density (violin shap) and median value (horizontal lines) for NC vs. PIH. The left half (red) of each plot represents the NC group, and the right half (blue) represents the PIH group. Asterisk (*) represents a significant difference in cytokine expression in the first-trimester pregnancy serum between the two groups.

To explore serum markers with potential predictive/diagnostic value for PIH in the first trimester of ART pregnancy, 48 cytokine expression levels were detected in patients with PIH and NC. PCA showed that the NC and PIH groups could be well distinguished by the first principal component (PC1), and a variety of cytokines contributed to PC1 and served as the main reason for the variation (**Figure 1A**). Heatmap and cluster analysis showed that the expression level of first-trimester serum cytokines in the PIH group was lower than that in the NC group (**Figure 1B**). In total, we found 29 differentially expressed cytokines between the two groups: IL-1β, IL-1α, IL-2, IL-2Rα, IL-4, IL-5, IL-6, IL-7, IL-9, IL-12 (p40), IL-12 (p70), IL-16, IL-17A, TNF-α, M-CSF, GM-CSF, LIF, SCF, Eotaxin, MIP-1β, FGF basic, MCP-1 (MCAF), β-NGF, SDF-1α, PDGF-BB, GRO-α, HGF, MIG, and TRAIL (**Figure 1C** and **Table 3**). Notably, compared with the NC group, the expression levels of all these cytokines were significantly lower in the PIH group (*P* < 0.05). In addition, we generated ROC curves for every cytokine that showed a significant difference (**Supplementary Figure 1**).

**Table 3:** Cytokine profile in first-trimester serum in ART pregnancy.

Cytokine (pg/mL)	Controls (N = 33)	PIH (N = 33)	*P* Value	Cytokine (pg/mL)	Controls (N = 33)	PIH (N = 33)	*P* Value
*IL-1β	2.93 (1.48, 6.24)	1.21 (0.82,3.57)	0.011	G-CSF	615.19 (191.61, 866.13)	399.05 (180.65,654.47)	0.132
*IL-1α	19.14 (12.91, 27.63)	12.91 (5.90,18.10)	0.001	*M-CSF	13.26 (10.39, 17.04)	9.78 (8.06,12.48)	< 0.001
IL-1ra	754.61 (563.53, 931.41)	621.73 (490.48,806.13)	0.132	*GM-CSF	3.97 (2.97, 5.02)	2.18 (1.62,2.97)	< 0.001
*IL-2	4.79 (3.33, 6.23)	2.78 (1.13,4.34)	< 0.001	*LIF	35.99 (23.57, 48.17)	19.35 (10.74,35.99)	0.005
*IL-2Rα	43.69 (32.21, 62.07)	30.92 (24.50,38.92)	< 0.001	*SCF	75.37 (56.95, 87.72)	54.54 (41.27,68.84)	< 0.001
IL-3	0.21 (0.04, 0.42)	0.18 (0.06,0.44)	0.746	VEGF	241.12 (147.15, 342.22)	231.61 (84.03,272.58)	0.197
*IL-4	3.70 (2.89, 5.22)	2.66 (1.64,3.77)	0.002	*Eotaxin	41.31 (32.02, 54.46)	32.69 (22.51,39.91)	0.001
*IL-5	64.32 (26.94, 126.01)	14.91 (8.41,58.69)	0.007	MIP-1α	68.56 (10.83, 106.22)	34.84 (9.81,71.25)	0.099
*IL-6	12.51 (6.06, 29.49)	5.24 (2.44,14.71)	0.002	*MIP-1β	388.30 (262.23, 529.08)	294.13 (234.93,379.84)	0.029
*IL-7	9.96 (5.51, 12.08)	5.51 (3.07,7.78)	< 0.001	*FGF basic	33.27 (25.93, 41.14)	25.94 (18.56,33.76)	0.03
IL-8	230.33 (36.70, 408.97)	86.52 (21.61,207.12)	0.052	*MCP-1 (MCAF)	52.70 (38.31, 73.37)	38.95 (24.46,52.13)	0.014
*IL-9	494.05 (452.60, 537.36)	462.89 (438.57,499.35)	0.022	MCP-3	1.83 (1.35, 3.49)	1.42 (0.91,1.96)	0.116
IL-10	1.68 (0.92, 3.14)	0.73 (0.17,1.68)	0.188	*β-NGF	41.57 (29.74, 46.19)	25.47 (17.27,36.90)	0.004
*IL-12 (p40)	64.38 (52.06, 93.65)	44.61 (34.61,54.53)	< 0.001	RANTES	1624.35 (13493.87, 21797.75)	1388.68 (11416.12, 18478.29)	0.095
*IL-12 (p70)	4.37 (2.48, 6.53)	2.28 (1.67,3.95)	0.01	*SDF-1α	2652.52 (2290.04, 2996.44)	2215.57 (1867.89,2593.89)	0.007
IL-13	0.86 (0.63, 1.45)	0.68 (0.28,1.04)	0.176	*PDGF-BB	7927.44 (5971, 9627.23)	6115.82 (4231.42, 7319.49)	0.031
IL-15	290.99 (166.36, 350.68)	198.59 (157.51,265.26)	0.164	*GRO-α	1517.16 (1367.91, 1618.30)	1377.92 (1269.18,1521.83)	0.022
*IL-16	87.15 (70.04, 123.29)	62.67 (43.89,110.71)	0.041	*HGF	384.31 (271.83, 493.41)	229.67 (189.72,348.35)	0.002
*IL-17A	6.66 (4.43, 8.54)	3.69 (2.96,6.66)	0.003	IP-10	622.64 (439.78, 779.46)	473.83 (386.64,608.99)	0.054
IL-18	42.53 (29.65, 55.84)	36.53 (25.78,48.17)	0.14	CTACK	501.42 (405.36, 715.20)	476.80 (400.04,550.13)	0.197
IFN-α2	8.22 (6.66, 10.26)	7.53 (5.42,8.90)	0.176	MIF	2552.33 (1572.57, 3226.17)	1720.26 (1015.06,2776.20)	0.106
IFN-γ	10.16 (7.21, 12.61)	9.35 (4.77,11.33)	0.4	*MIG	169.31 (135.75, 237.82)	109.68 (72.98,160.12)	< 0.001
*TNF-α	56.98 (45.56, 76.22)	41.73 (29.39,54.70)	0.003	SCGF-β	1203.12 (106619.85, 136337.81)	1127.04 (99055.56, 129400.67)	0.108
TNF-β	761.20 (687.85, 796.32)	729.31 (662.35,768.62)	0.064	*TRAIL	29.92 (21.70, 39.14)	21.70 (18.06,27.57)	0.01

Values are median (P25, P75). P < 0.05 was considered statistically significant and indicated by an asterisk.

### Relationship Between the First-Trimester Serum Cytokine Profile and Subsequent PIH

**Figure 2:**
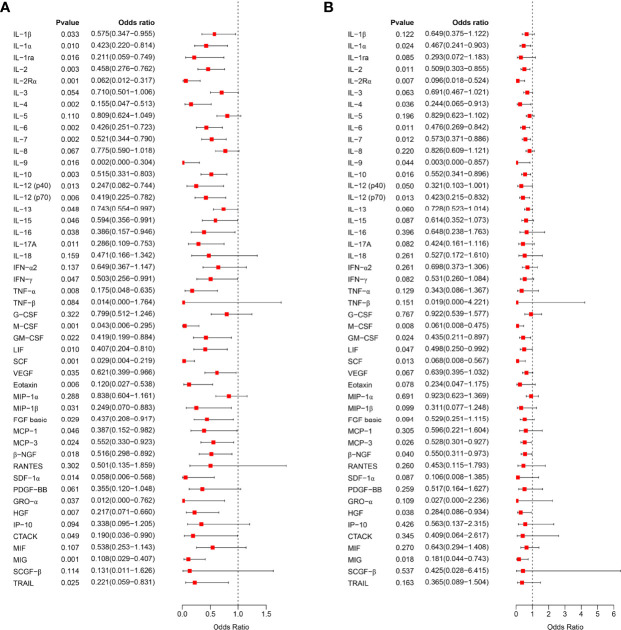
Correlation analysis of early pregnancy serum cytokines and PIH. **(A)**, The relationship between the expression level of 48 cytokines in early pregnancy serum and the occurrence of PIH in later pregnancy was analyzed by univariate logistic regression analysis and visualized as a forest map. **(B)**, After correcting the mean arterial pressure before pregnancy, the relationship between the expression level of 48 serum cytokines in early pregnancy and the occurrence of PIH in later pregnancy was analyzed by logistic regression analysis. OR > 1 indicates a risk factor, and OR < 1 indicates a protective factor.

We further analyzed the relationship between serum cytokine levels in the first trimester of pregnancy and the occurrence of PIH in later pregnancy by logistic regression analysis. Univariate logistic regression analysis showed that 35 cytokines were associated with subsequent PIH (**Figure 2A**). After adjusting for the influence of prepregnancy mean arterial pressure, 17 cytokines in early pregnancy serum were found to be negatively associated with an increased risk of PIH in later pregnancy, including IL-2Rα, M-CSF, IL-6, IL-2, β-NGF, IL-7, IL-12 (p70), SCF, IL-10, IL-9, MIG, GM-CSF, LIF, IL-1α, MCP-3, IL-4, and HGF (**Figure 2B**).

### Predictive Value of Serum Cytokines in Early Pregnancy for PIH in ART Pregnancy

**Figure 3:**
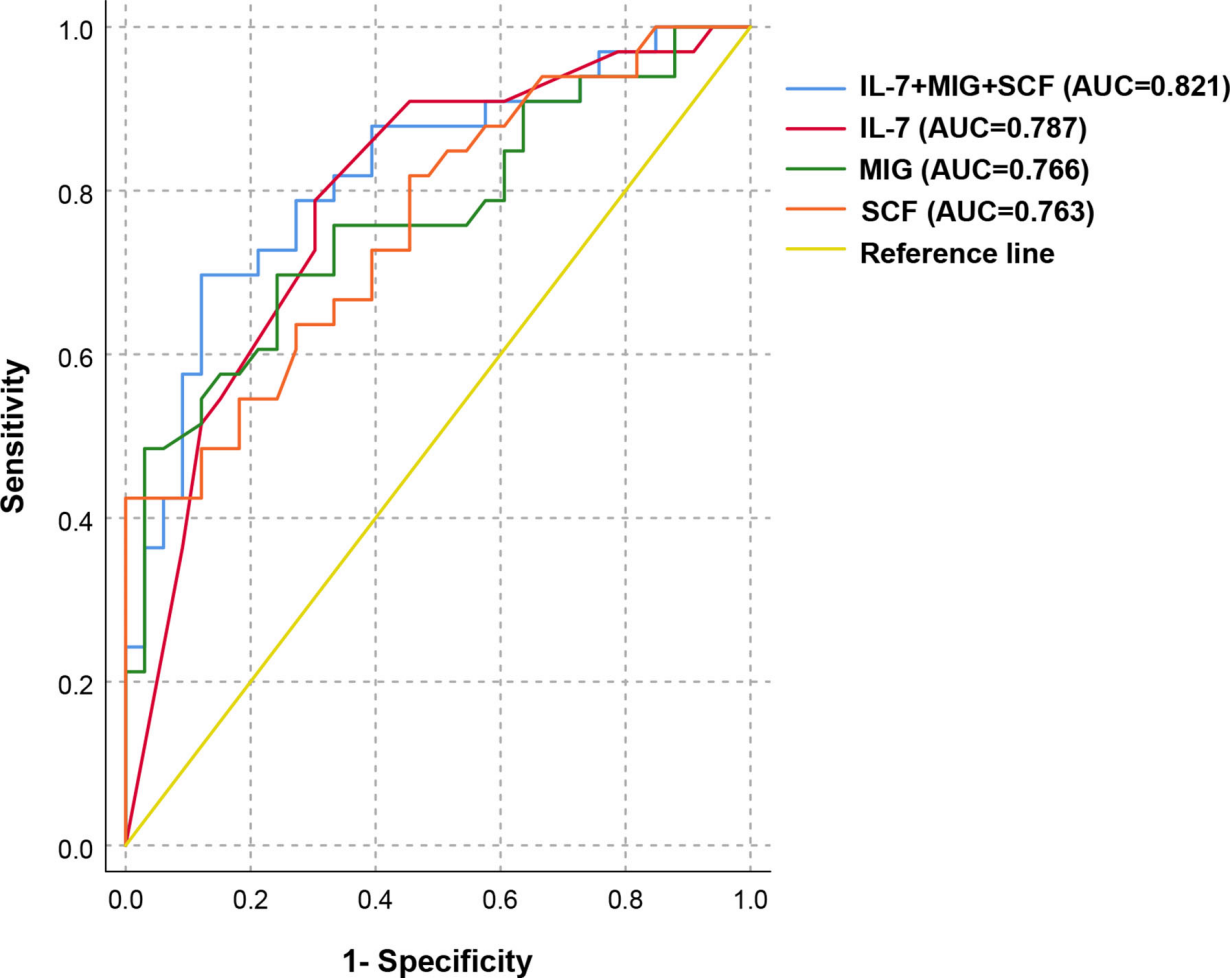
ROC analysis of the combined prediction model for the prediction of PIH in ART pregnancy. ROC curves show the results of ROC analysis for differentiation between PIH and NC in ART pregnancy. ROC curve analysis revealed that IL-7, MIG, and SCF appeared to be potential biomarkers for the separation between PIH and NC in the first trimester of pregnancy (AUC = 0.821, 95% CI: 0.718-0.924, *P* < 0.001), with a sensitivity of 81.8% and a specificity of 72.7%.

We selected the above 17 cytokines related to PIH and further explored their predictive value as potential target molecules for PIH. The ROC curve analysis results are shown in **Table 4**. Among the above 17 PIH-related cytokines, IL-7, MIG and SCF are the top three cytokines with pronounced predictive values for PIH. We then selected IL-7, MIG, and SCF to construct a combined prediction model, which was found to have good predictive value for PIH. (AUC 0.821, 95% CI: 0.718-0.924, *P* < 0.001) (**Figure 3**). The AUC of the combination of IL-7, MIG, and SCF was larger than that of every single cytokine, which proved that the combined prediction model had a better ability to predict PIH than other individual indicators. The sensitivity (true-positive rate) was 81.8%, and the specificity (true-negative rate) was 72.7%. Other analyses for variable selection and the suboptimal combined prediction model are graphically represented in **Supplementary Table 1** and **Supplementary Figure 2**. In addition, we evaluated the predictive value of decreased cytokine levels on pregnancy outcome, including delivery mode, delivery at <37wk, and low birth weight infants. We found IL-2Rα, M-CSF, SCF, and MIG are individual risk factors for cesarean delivery, and a combination of these four risk factors provide a good predictive value (AUC=0.722, *P* = 0.004) for cesarean delivery mode (**Supplementary Figure 3**).

**Table 4:** ROC analysis of serum cytokines in early pregnancy for PIH predictive/diagnostic value.

Cytokines	AUC (95% CI)	*P* value
IL-7	0.787 (0.676-0.899)	< 0.001
MIG	0.766 (0.651-0.882)	< 0.001
SCF	0.763 (0.649-0.876)	< 0.001
M-CSF	0.759 (0.644-0.875)	< 0.001
GM-CSF	0.759 (0.637-0.881)	< 0.001
IL-2	0.758 (0.641-0.876)	< 0.001
IL-2Rα	0.756 (0.64-0.873)	< 0.001
IL-6	0.737 (0.617-0.857)	0.001
IL-4	0.736 (0.616-0.857)	0.001
IL-12 (p70)	0.729 (0.607-0.852)	0.001
β-NGF	0.729 (0.607-0.850)	0.001
LIF	0.723 (0.599-0.847)	0.002
HGF	0.721 (0.594-0.849)	0.002
IL-1α	0.716 (0.593-0.839)	0.003
IL-10	0.697 (0.567-0.826)	0.006
MCP-3	0.675 (0.545-0.805)	0.015
IL-9	0.664 (0.533-0.796)	0.022

AUC: area under the curve; CI: confidence interval. Significant difference (P < 0.05).

## Discussion

PIH is a common pregnancy-specific complication that threatens both maternal and fetal safety (18–20). As a result of the lack of definitive therapies except for early delivery of the feto-placental unit once PIH occurs, in recent years, guidelines have placed greater emphasis on early warning, early detection and early intervention (21–23). Low-dose aspirin (LDA) is suggested to be used beginning from 11 gestational weeks for better prevention efficacy in high-risk women (13, 23–25). In regard to ART pregnancy, the evaluation and prediction methods of PIH remain to be elucidated. In this study, we brought forward the serum cytokine testing time to as early as 11-13 gestational weeks for a potential timely prediction for PIH after ART. It is worth noting that even the routinely used predictive parameters, such as Activin A, PlGF, sFlt1 and sFlt1/PlGF, were not able to differ PIH from controls in ART women, which is in line with another recent study targeting the IVF population, reporting no significant difference in first-trimester serum Activin A between normotensive controls and pregnancies with subsequent PE/PIH (26). This is possibly ascribed to the potentially undefined population specificity, emphasizing the importance of exploring novel biomarkers for PIH in the ART population. Our results show that the cytokine profile in the first trimester of ART pregnancy demonstrated a significantly downregulated trend in ART women destined to develop PIH. We found 17 cytokines in the serum of first-trimester pregnancy related to the increased risk of PIH, including 10 interleukins (IL): IL-1α, IL-2Rα, IL-2, IL-4, IL-6, LIF, IL-7, IL-9, IL-10, and IL-12 (p70); three colony stimulating factors (CSF): M-CSF, GM-CSF, and SCF; two chemokines: MIG and MCP-3; and two growth factors (GF): β-NGF and HGF. Moreover, we constructed a first-trimester prediction model using IL-7, MIG, and SCF for screening out the cases at high risk of developing PIH preceding clinical manifestations in ART pregnancy.

With increasing knowledge, the antecedents of poor placentation of PIH are considered to be immunological in origin (14, 27, 28). At the maternal-fetal interface during early pregnancy, the adaptive regulation of maternal immunity is mainly characterized by the transformation of T helper type 1 (Th1) cytokines to Th2 cytokines (29, 30). Studies have shown that the shift toward Th2 may be hindered in PIH, with the cytokine profile in peripheral blood being mainly Th1 (31, 32). Our results support this theory; that is, IL-4, IL-9 and IL-10, which are related to Th2 in the serum of PIH patients during early pregnancy, are significantly downregulated.

The cytokine profile of PIH in previous studies was usually demonstrated during middle and late pregnancy, which is generally characterized by a pro-inflammatory state, showing a higher level of proinflammatory cytokines, such as IL-1, IL-6, IL-8, IL-17, and TNF-α, and a lower level of anti-inflammatory interleukins, especially IL-10, IL-33, and IL-35 (15, 33–35). Compared to the pro-inflammatory state in middle and late pregnancy, our assay in the ART population showed that proinflammatory factors such as IL-6 and IL-12 were downregulated in the first-trimester serum of PIH patients. A study in rats indicated that IL-6-mediated arterial pressure elevation is due to a response to chronic reductions in uterine perfusion pressure during pregnancy (36). Therefore, we hypothesized that in ART pregnancy, the shift toward a proinflammatory phenotype in PIH probably does not occur in the very early stages of the disease. Similarly, the association of IL-12 serum levels with the development of PIH has been assessed in various studies, drawing no certain conclusions because of conflicting evidence (37–41). In fact, a proinflammatory phenotype is the result of compensation for insufficient immune activation and early placental establishment in the first trimester. Therefore, most studies devoted to finding biomarkers in middle and late pregnancy to predict PIH failed to achieve good clinical application effects (42). The difference in serum proinflammatory factors in PIH patients at different pregnancy stages and the dynamic changes need to be further explored.

In spontaneous pregnancy, semen exposure between coitus and conception primes innate and adaptive immune cells for prepared maternal immune tolerance in advance of potential implantation (43–45). In contrast, ART pregnancy tends to directly transfer embryos under the premise of prohibiting sexual intercourse in the transfer cycle. This may at least partially explain why ART is associated with an increased risk of PIH and at the same time support our results that the PIH group showed lower serum cytokine levels than the normotensive group at 11-13 weeks gestation. In other words, inadequate tolerance induction before embryo transfer results in a universally low level of serum cytokines in the first trimester of pregnancy, which may disrupt the inflammatory process of embryo implantation and is in turn involved in the shallow invasion of trophoblasts in early PIH (46).

The present study is based on the human biobank of our large-scale assisted reproductive cohort platform and is the first to delineate the first-trimester serum cytokine profile of PIH, especially for the ART population. We found that maternal serum concentrations of multiple immune-related cytokines were significantly decreased in the first trimester of ART pregnancy with subsequent PIH. With conjoint analysis of serum IL-7, MIG and SCF, we demonstrated a first-trimester prediction model before PIH onset and subsequent compensatory response. Admittedly, the number of participants included in the current study is still limited and further research with a large sample size is needed to support our findings.

The establishment and maintenance of pregnancy poses great challenges to the maternal immune system. Uncoordinated maternal immunization, especially in the first trimester of pregnancy, will be a potential cause of a series of pregnancy complications (47, 48). Currently, immunotherapy has been applied to the treatment of a variety of reproductive diseases, such as recurrent spontaneous abortion (RSA) and recurrent implantation failure (RIF) (49), while the immunotherapy against PIH still needs a long way to go. Our study described the first-trimester serum cytokine profile in pregnancies conceived after ART and provided a better understanding of the immunological etiology and pathophysiology of PIH in the specific population. Additionally, the first-trimester immune disturbance enables early prediction, which guarantees enough time for low-dose aspirin (LDA) prevention before the appearance of hypertension symptom and hopefully may also promote novel therapeutic strategies on immune regulation and shed light on the clinical management of PIH in the ART population.

## Data Availability Statement

The original contributions presented in the study are included in the article/**Supplementary Material**. Further inquiries can be directed to the corresponding authors.

## Ethics Statement

The studies involving human participants were reviewed and approved by Ethical Committee of Reproductive Medicine of Shandong University. The patients/participants provided their written informed consent to participate in this study. Written informed consent was obtained from the individual(s) for the publication of any potentially identifiable images or data included in this article.

## Author Contributions

YL conceived and designed the project. XL, LG, and SZ performed the experiments and analyzed the data. XL, YC, and YN collected the clinical serum samples and information. XL, LG, SZ, SH, and ZL wrote the manuscript. YL and JY critically revised the manuscript. All authors have been involved in interpreting the data and approved the final version.

## Funding

This study was supported by grants from the National Key Research and Development Program of China (2021YFC2700604), the National Natural Science Foundation of China (82101784, 82171648), the Key Research and Development Program of Shandong Province (2021LCZX02), the Natural Science Foundation of Shandong Province (ZR2020QH051), the Natural Science Foundation of Jiangsu Province (BK20200223), Taishan Scholars Program for Young Experts of Shandong Province (tsqn201812154) and the Young Scholars Program of Shandong University.

## Conflict of Interest

The authors declare that the research was conducted in the absence of any commercial or financial relationships that could be construed as a potential conflict of interest.

## Publisher’s Note

All claims expressed in this article are solely those of the authors and do not necessarily represent those of their affiliated organizations, or those of the publisher, the editors and the reviewers. Any product that may be evaluated in this article, or claim that may be made by its manufacturer, is not guaranteed or endorsed by the publisher.
