# Supplementary material for: First-Trimester Serum Cytokine Profile in Pregnancies Conceived After Assisted Reproductive Technology (ART) With Subsequent Pregnancy-Induced Hypertension
Source: Front Immunol. 2022 Jul 1;13:930582. doi: 10.3389/fimmu.2022.930582 (PMC9283642; doi:10.3389/fimmu.2022.930582)

Supplementary Material

**Supplementary Table 1.** Variables included in the multivariate binary logistic regression model

|  | **S.E.** | ***P*** | **OR (95% CI)** |
| --- | --- | --- | --- |
| SCF | 1.075 | 0.003 | 0.042 (0.005-0.346) |
| IL-12 (p70) | 0.317 | 0.036 | 0.514 (0.276-0.957) |

Abbreviations: S.E.: standard error; OR: odds ratio; CI: confidence interval.

**Supplementary Figure 1.** ROC analysis with the area under the curve (AUC) of the 29 cytokines with significant differences between PIH and NC in ART pregnancy.


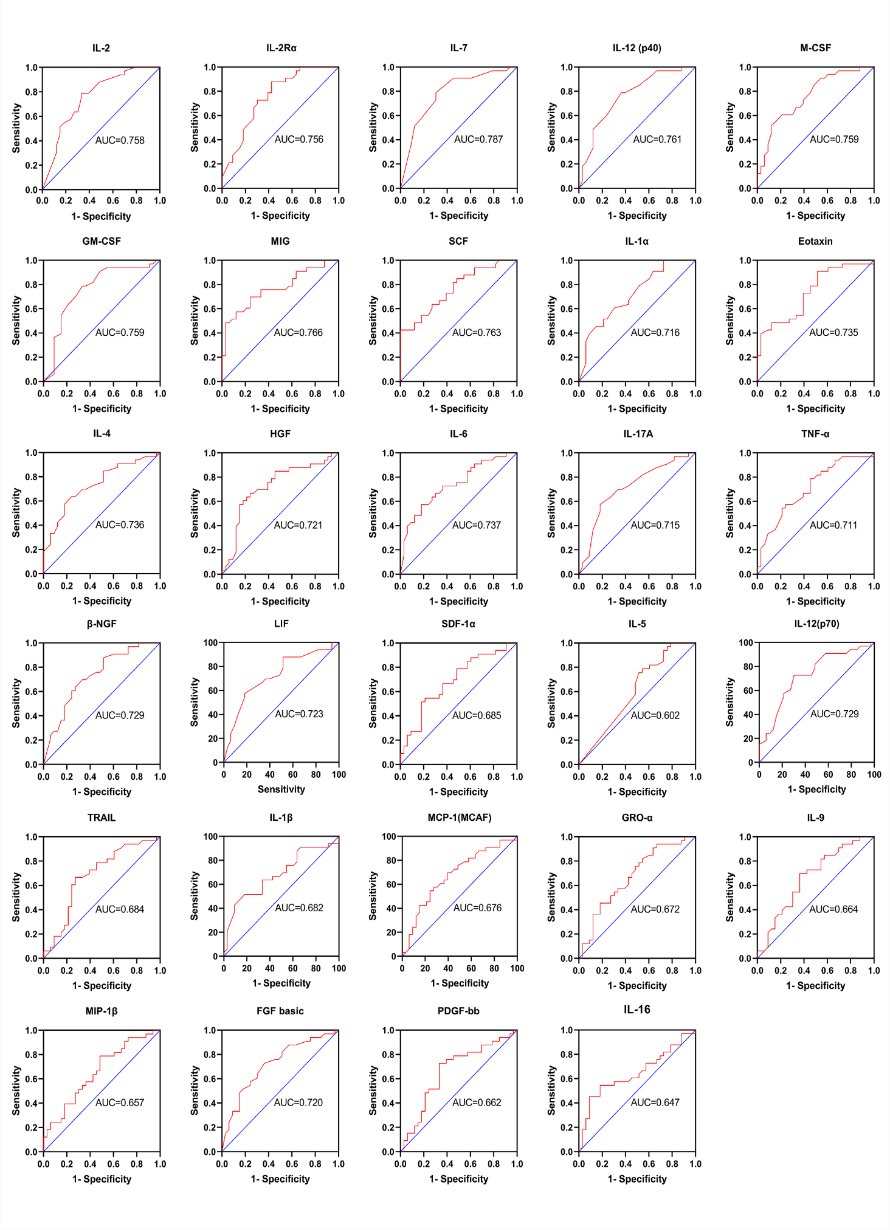


**Supplementary Figure 2.** ROC analysis of the two combined prediction models with different variables for the prediction of PIH in ART pregnancy.

ROC analysis shows that the AUC of the combination of SCF and IL-12 (p70) is bigger than that of SCF or IL-12 (p70) alone, but smaller than the AUC of the combination of IL-7, MIG, and SCF.


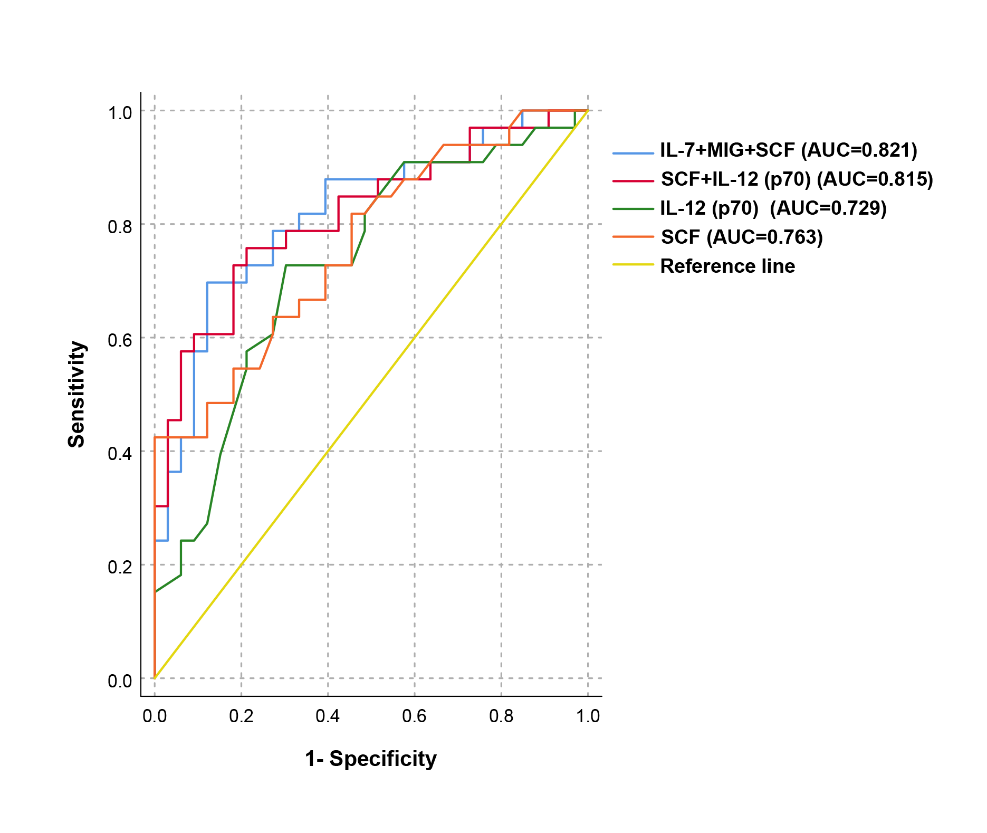


**Supplementary Figure 3.** ROC analysis of the first-trimester cytokines for the prediction of cesarean delivery in ART pregnancy.


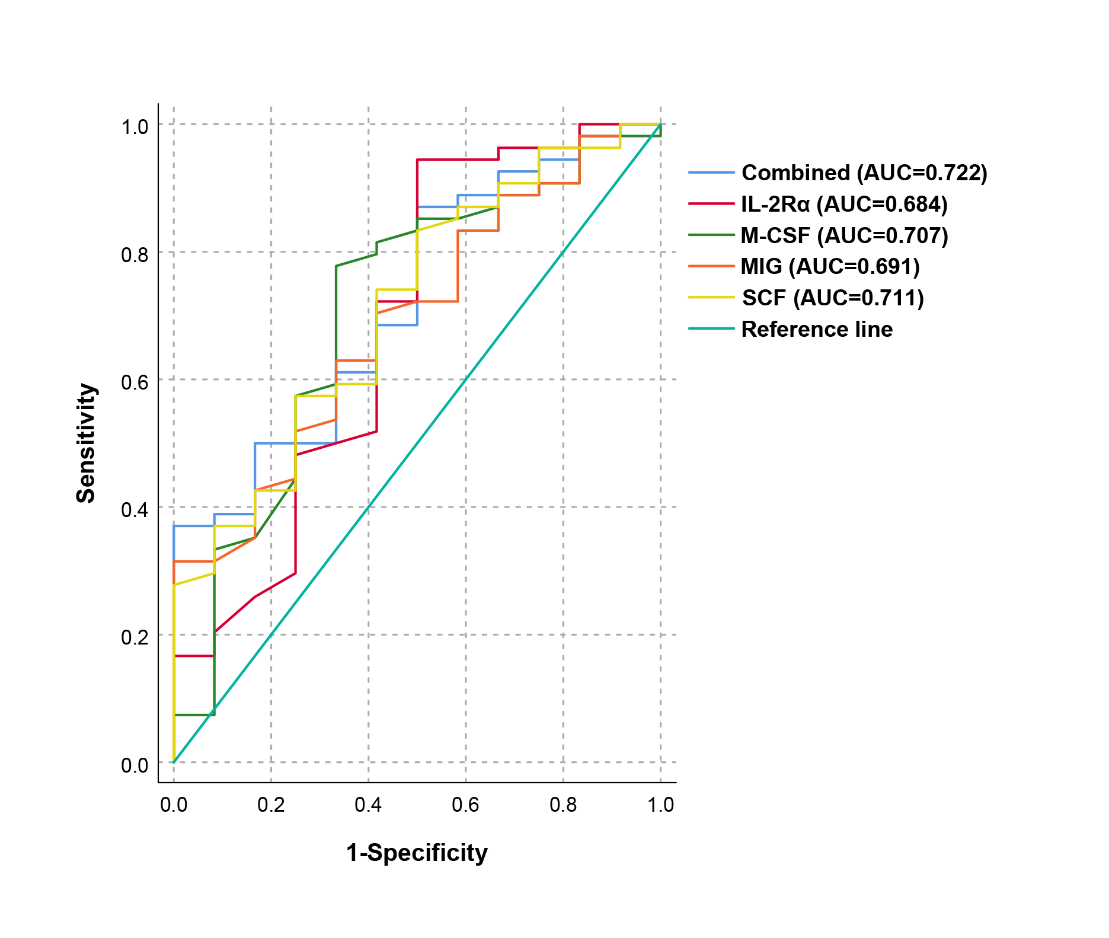

Supplement: Supplementary file 1 [file DataSheet_1.docx]
